# Next generation transcriptomes for next generation genomes using *est2assembly*

**DOI:** 10.1186/1471-2105-10-447

**Published:** 2009-12-24

**Authors:** Alexie Papanicolaou, Remo Stierli, Richard H ffrench-Constant, David G Heckel

**Affiliations:** 1 Department of Entomology, Max Planck Institute for Chemical Ecology, Jena, Germany; 2 School of Biological Sciences, Centre for Ecology and Conservation, University of Exeter, Penryn, UK; 3 Department of Computer Science and Statistics, University of Rhode Island, Kingston, USA

## Abstract

**Background:**

The decreasing costs of capillary-based Sanger sequencing and next generation technologies, such as 454 pyrosequencing, have prompted an explosion of transcriptome projects in non-model species, where even shallow sequencing of transcriptomes can now be used to examine a range of research questions. This rapid growth in data has outstripped the ability of researchers working on non-model species to analyze and mine transcriptome data efficiently.

**Results:**

Here we present a semi-automated platform '*est2assembly*' that processes raw sequence data from Sanger or 454 sequencing into a hybrid *de-novo *assembly, annotates it and produces GMOD compatible output, including a SeqFeature database suitable for GBrowse. Users are able to parameterize assembler variables, judge assembly quality and determine the optimal assembly for their specific needs. We used *est2assembly *to process *Drosophila *and *Bicyclus *public Sanger EST data and then compared them to published 454 data as well as eight new insect transcriptome collections.

**Conclusions:**

Analysis of such a wide variety of data allows us to understand how these new technologies can assist EST project design. We determine that assembler parameterization is as essential as standardized methods to judge the output of ESTs projects. Further, even shallow sequencing using 454 produces sufficient data to be of wide use to the community. *est2assembly *is an important tool to assist manual curation for gene models, an important resource in their own right but especially for species which are due to acquire a genome project using Next Generation Sequencing.

## Background

Much of the recent progress in our understanding of genomics has come from the study of model genetic organisms such as the fruit fly *Drosophila melanogaster*, the nematode worm *Caenorhabditis elegans *and the zebrafish *Danio rerio *[1]. In these model species, a full genome sequence combined with a well annotated collection of gene models is currently available. To address fundamental questions in evolutionary biology, however, we need to expand the genomic and transcriptomic resources available for non-model species, notably insects [1]. Non-model insects represent attractive models for the study of a range of important biological questions both of applied and fundamental importance, such as those relating to studying insecticide resistance [2], wing pattern development [3] or co-evolution [4]. From a functional biologist's point of view, crucial experimental tractability can be gained via a combination of rich sequence data (including Expressed Sequence Tag - EST - collections from several tissues), gene models, functional annotation and in-depth knowledge of an organism's genetics, preferably coupled with the ability to manipulate them [5,6].

Since the advent of EST sequencing, the number of organisms represented in dbEST (which houses all public Sanger-sequenced EST projects) [7] has exploded. Transcriptomics has grown to be at least as an important resource to non-model species communities as it has proven for the traditional models. It is now conceivable that many non-model species will have at least a workable outline of their genomes available. In turn, large collections of ESTs important in genome annotation will be generated as the community identifies -omics as a major resource for species with an evolutionary importance [8-10]. This scenario is already a reality as the related technologies become more cost-effective. Such non-model species transcriptome projects, being focused on particular applications and biological questions, are especially useful to the wider community even if not targeting annotation of any specific genome [11-13]. One of the most important benefits of shallow EST sequencing is the ability to acquire candidate sequence data for downstream applications such as phylogenetics, multi-locus population genetics and expression studies [14]. It is hoped that with a wide application of Next Generation Sequencing (NGS) the bottleneck in obtaining sequence data will no longer exist [15]. The reality is, however, that this vast amount of sequence data has outstripped the ability of most researchers working on non-model species, who often have limited bioinformatic support, to analyze and mine their new datasets. Further, there is currently no standardized platform for providing researchers with such analyses for transcriptomes. The Generic Model Organism Database (GMOD) group, derived mainly from the model species communities, is a collection of software, platforms and standardized approaches in dealing with -omic data. They are best known for the GBrowse project, the sequence viewer used by WormBase, FlyBase and others [16]. One of the most important contributions of the GMOD group, however, is the development and dissemination of standards capable of generic use: extensive use of BioPerl [17], database connectivity frameworks and Chado, the generic database schema for almost any type of data produced by the community [18]. Such standards allow tools to be capable of a unique level of interconnectivity and interoperability.

Here we describe a software suite, *est2assembly*, which aims to address the above bioinformatic deficit while being embedded in the GMOD framework. It accepts raw sequence data (from 454 or Sanger technologies) and produces annotated assemblies in a GMOD/Chado-compatible format with minimum user input. Further, using the common file format of GFF (which stands for General Feature Format), users can share their data with collaborators and visualize them with tools such as GBrowse. The platform is highly automated and standardized, and we show it allows for direct comparison between various datasets. The modular nature of *est2assembly *allows users to independently make use of different subroutines. Extensive log files guide the user through the assembly process and the output. We demonstrate this platform using a range of 454 data from a phylogenetically diverse sample of insects. We benchmark the platform and compare these non-model species collections with 745,124 public EST data from *D. melanogaster *collected via conventional capillary sequencing which is still considered the gold standard in insect EST data and *Bicyclus anynana*, the butterfly with the highest number of Sanger-sequenced ESTs in GenBank.

## Implementation

### Software dependencies

All software on which the platform depends is free and installation requirements are straightforward. In brief, the platform makes extensive use of BioPerl (1.6+) and other open-source Perl modules available from CPAN. The GPL-licensed MIRA assembler is required [19]. The proprietary Newbler2 - including the associated SFF toolkit - (454 Life Sciences) is optional. For 454 next generation sequencing files we use sff_extract [20] as this is the only known 454 basecalling software which is not under a restrictive license. In this paper, we made use of the complete platform and utilized both Newbler2 and MIRA version 2.9.37. Further, some modules have dependencies such as installation of the Chado database schema [18], EMBOSS [21], NCBI-BLAST [22], SSAHA2 [23], RepeatMasker [24] (with an recommended registration to RepBase [25]), prot4EST [26], FASTY 3.4 [27] and annot8r [28]. Due to the modular nature of the platform, a researcher needs to install only the components which will be of use their application of the platform. We provide a comprehensive installation script to ease the procedure of installation of the above 3^rd ^party software.

### Read pre-processing

The est_process module is driven by *preprocessest.pl *which accomplishes the following steps: i) project creation, including calling the bases or reading the SSF files; ii) masking short sequences (e.g. adaptors) using SSAHA2; iii) BLAST2 to detect unwanted sequences (as defined by the user) which can cause problems in the assembly (e.g. mitochondrial, rDNA and contaminants); iv) removal or masking using these BLAST output files; v) Repeat masking performed using RepeatMasker and a user provided database (which can be extracted from RepBase and customized); vi) polyA/T screening performed using a tiered approach of a custom algorithm. Then a final step cleans the output files and prepares assembly specific input files including an XML NCBI TraceInfo file. A user may interrupt the program and resume from any of the above steps.

The conversion of raw trace files to input files for an assembly is performed independently for each technology. Users have the option to convert and quality trim Sanger-derived sequences using the gold standard of phred or in the case of data derived from ABI sequencers data, make use of any internal KB basecalling. In the latter case, we use a custom quality trim subroutine provided by Steffi Gebauer-Jung (MPI for Chemical Ecology) involving two sliding windows to avoid local optima: a larger one scans the trace for a sudden drop in quality values and a finer search pinpoints the exact location. For 454 sequencing, due to licensing prohibiting the use of Roche's proprietary flowgram extracting software by ordinary researchers, SFF files are extracted using sff_extract, an open-source alternative. In addition to detection of low quality regions, we identify adaptor sequence introduced either in the making of the cDNA library or subsequent pre-sequencing steps. We use a two tiered search using SSAHA2, which combines the SSAHA and the cross_match algorithms [29] and BLAST2 from NCBI. We found that the SSAHA2 search itself is best utilized by using three iterations: two searches for adaptor sequence (with one prior- and one post-polyA/T masking) and one restriction site search with a parameterization for extremely short target sequences (restriction sites tend to be ca 7-10 bp). The platform uses BLAST2 to screen for common contaminants found in molecular biology labs via a customizable FASTA database. In any transcriptome project, it is also undesirable to clutter the assembler with sequences that are overrepresented due to transposon activity, mitochondrial or rDNA origin. The platform allows users to screen them using RepeatMasker via a user-defined database. To assist Lepidopterists in particular, we have included a prediction of repetitive elements from *Bombyx mori *(C. Smith, University of San Francisco, pers. communication) which users can concatenate with the Insect repeat library from RepBase. The intensive steps of running BLAST and SSAHA2 can seamlessly utilize multiple threads to reduce run time.

Trimming of polyA/T tails is essential in EST projects and we use a routine to iteratively scan for such homo-oligomers. The routine can also be used independently of the platform and is highly customizable; users can specify which base (A, T, C or G) they wish to search for, seed length, min/max length of homo-oligomer, depth of search of each sequence and other options. In order to minimize false positives in A/T rich genomes or errors produced by the pyrosequencing methodology of 454, the platform utilizes 5 rounds with increasing minimum length and decreasing search seed length. Except for the second round pair, the scan is performed only at the ends of the sequence for a length of one-third of the total sequence length. The second round scans deeper to 350 bp from each end. An additional feature of the routine is the use of any suspected polyadenylation signal site (PAS) upstream of a hypothesized polyA/T site. Currently, we use a simple pattern search but implementing a model-based approach [30] could be of use. This assists in correcting the masking and avoiding over-trimming of the 3' UTR or for allowing a short polyA sequence which would normally be below acceptable length to be masked. In addition, it uses this information to detect false positives if there is a significant amount of sequence (>50 bp) between the end of the polyA and the start of any vector masked sequence (or the end of the sequence). If no PAS site is found upstream of the polyA tail (in a 50 bp window) and the suspected polyA is shorter than the specified cut-off, then the polyA is not masked but still tagged in the log-files. We found that this option enhances polyA masking in Sanger-derived sequences but by default is switched off for short reads. An output for each polyA/T found and which criteria were used is produced in a log file should users wish to exploit them in gene model construction. At this stage, an XML file (using the NCBI TraceArchive template) containing the low quality, adaptor sequence and polyA/T trim points is generated. This file, when used with the original untrimmed and unmasked file, can guide assemblers such as MIRA on how to perform clipping of undesired regions. This approach can be used to tackle any potential false positives that may arise in the preprocessing steps.

It is worth noting here that we find that near-perfect signatures of the 454 adaptor sequences can persist even within regions of high quality assembly, which could be the result of the chimeric ligation of molecules. In such a scenario, we recommend that if manual inspection is not feasible that the sequence region is masked and the assembler allowed to determine if the region is truly an adaptor sequence or part of the sequenced species genome: false positives will have multiple reads in the assembly exhibiting high identity down- and up-stream of the suspected site and therefore still assemble. To facilitate assemblers who cannot make such judgements, preprocessest.pl allows the flagging of sequences which have more adaptor sequences than the user defines. If the user wishes then only the longest stretch of high quality sequence between two adaptors is used.

### Optimal assembly

The output files of est_process are provided to the second module, parameterize_assembly. It could be straightforward to plug-in various assemblers in future versions but we currently make use of Newbler as it is the standard for 454 data and MIRA due to its ability to analyze Sanger/Next Generation data concurrently and the provision of excellent support. In the current version, datasets from Sanger and/or 454 can be provided and users will concatenate any datasets originating from identical technologies. A configuration file is responsible for defining which parameters are passed on to the assembler MIRA. This allows for multiple runs of the same datasets in order to explore the parameter space.

Assembly quality is estimated using analyze_blast.pl via summary indexes based on the coverage of one or more reference organism databases used in a similarity search (e.g. NCBI-BLAST). Coverage is calculated on a base pair basis by counting unique hits to a particular base pair. Overlapping coverage (i.e. redundancy) is calculated by counting the total number of hits a base pair (or amino acid; the platform uses the term position) receives. We perform these calculations for both the assembly and the database. The ratio of redundancy over coverage is summed for the database and the assembly. If more than one reference database is used (which is recommended in order to discount any organism-specific effects) then the total sum is used. One has to be aware that the absolute numbers of coverage are volatile as they are dependent on the BLAST cut-offs used. When the same cutoffs are used, however, comparison of assemblies is a meaningful index to evaluate how parameterization influences the assembly. Another quality control index is the number of reads included in the assembly. This can act as a proxy for the downstream utility of an assembly, for example the number of SNPs which can be determined or the likelihood we can detect alternative splicing or frame-shift-causing sequencing errors. Finally, we also consider the proportion of the reference database covered (or the average if more than one is used) as a proxy to eventual gene finding.

In EST assemblies a large portion of the consensus can be non-coding sequence. Such sequences often diverge rapidly due to the lack of any selective constraints. The unfortunate result in any assembly process is that sequences of this type, which are from identical genomic regions, fail to assemble together. The script trim_assembly.pl is one of the two methods to remove such redundant contigs. It first scans for polyA/T tails which may have been built during the assembly. Then it defines a set of 'high-quality' contigs using user-specified cut-off for length and number of reads included. The other contigs are only included in the final set if they a) don't have a high sequence similarity to a high-quality contig, b) have a high sequence similarity to a reference proteome, c) specifically requested by the user by proving a list file. The output of the contigs which have been excluded is cataloged in a log file.

### Protein identification, SNP discovery and data mining

Data mining of EST projects is driven by searching the dataset for the signature of a favorite protein or sequence. The platform makes use of BLAST similarity searches using the contig consensus. The analyse_assembly.pl and analyze_blast.pl scripts, which are employed during parameterization, can be used standalone to estimate the quality indexes for any BLAST report. They allow users to identify the exact coverage of a FASTA input file in relation to a reference database using BLASTx, BLASTn or tBLASTx. They provide the ability to run multiple blasts in a threaded fashion with one command. The script has the additional ability to output a FASTA file with the part of the input file which matches the reference and a second file with the part which did not. This approach is very useful in extracting the segment of the assembly which is known to be coding. For example, a tBLASTx approach is useful when multiple species have been sequenced but no reference proteome exists or is under-annotated (see Results section).

Deeper annotation can be performed using predicted proteins. Protein predictions are accomplished using prot4EST [26] (included in the distribution). The current version of prot4EST does not produce the Open Reading Frame (ORF) which can differ substantially from the contig (one of the utilities of prot4EST is that it corrects for frameshifts). We acquire the ORF using FASTY or failing that, EMBOSS's transambig and attempt to correct for any ambiguous codons to match the consensus. These are then annotated using similarity to known proteins which may have annotated ontology terms: Gene Ontology (GO) [31], Enzyme Commission (EC) [32] and Kyoto Encyclopaedia of Genes and Genomes (KEGG) [33]. Annotations based on electronic similarity are assigned using annot8r. If computational resources allow, InterProScan annotations can be included to allow users to search for specific protein domains, motifs and sequence signals. The output of the above procedures is then converted to a common file format using ic_annot8r2gff.pl and can be databased.

Further, we provide the script *analyse_assembly.pl *to help with BLAST reports on a single computer but if EST projects are commonplace then access to a PC-farm or a high performance computing system is required. For this reason, we include a set of simple scripts to facilitate use and error-checking of LSF queue submissions allowing, therefore, for the automation of BLAST and InterProScan annotations.

Of particular interest to biologists is the identification of Single Nucleotide Polymorphism markers (SNPs). The assembler MIRA produces such a set and can be included in the assembly GFF file. We also extract a 'high-quality' SNP dataset by including those SNPs which have the minor allele frequency above a user-specified threshold and have at least 20 invariable bases up and downstream of the SNP position. This padding can be customized by the user but we default to 20 in order to design primers and create a unique identification sequence for submission to dbSNP [34]. This SNP identification is accomplished via ic_create_snps.pl which estimates the position of each SNP in relation to the ORF and, if determined as coding, provide the codon position, the alleles and whether the nucleotide change causes a synonymous or non-synonymous amino acid change. Due to prot4EST's approach to determine the ORF, a simple translation of co-ordinates is not possible. We, therefore, perform a local alignment using FASTY [27]. We also include a similar implementation for SEAN [35] which would be of use to users with small amounts of data.

**Figure 1:**
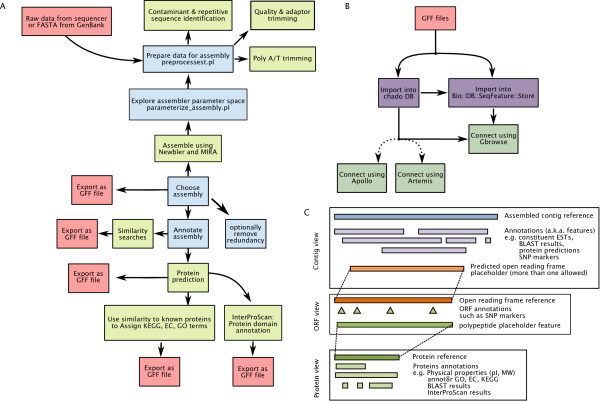
**Schematic diagram of the *est2assembly *platform**. (A) Sub-routines processing and annotating the EST data. Note that all outputs are in the common GFF standard and therefore can be accessed by GMOD-compatible software. (B) Diagram illustrating the the ability of *est2assembly *to produce a GBrowse sequence view. (C) Diagram illustrating a triple page approach to graphical outputs from *est2assembly*: First, a page showing the assembled contig and associated annotation, second, a page showing each predicted ORF and its annotation and, third, a page focused around the annotated protein object. Note that each page is linked and allows for rapid navigation to genes of interest.

We can utilize GFF as the middleman to populate Chado and Bio::DB::SeqFeature (SeqFeature) database schemas. In this paper, we focus on the simple format and speed advantages of SeqFeature as most users will be dealing with a limited number of datasets. We provide a set of scripts that produce both Chado- and SeqFeature-compatible GFF files for each data type which the platform is capable of processing: CAF assemblies in read-contig or contig-read sorting order, BLAST reports, ORF predictions, SNPs predictions and KEGG, GO and EC annotations from annot8r. As the proper linking of the various reference sequences and their features is essential, we have a specific strategy for creating the GFF files for use with GBrowse (Figure 1C): a contig view is composed of a reference contig and associated annotation features such as the assembled EST data, SNP markers, BLAST annotation etc. is anchored to it. The ORF feature is also anchored to the contig but also exists as a separate reference sequence in a second web page. This ORF object has its own associated annotation, including a polypeptide features which serves as an anchor for the protein prediction reference. Likewise, this protein prediction allows for anchoring protein-based annotations such as estimated molecular weight, assigned ontology terms and other protein-based data in a third web page. This approach enforces the one-to-one relationship between an ORF and a protein but allows for one-to-many relationships between an assembled contig and protein predictions.

### Benchmark datasets

Data for benchmarking the platform was provided by dbEST (for *D. melanogaster *and *B. anynana*) or by collaborators. In more detail, the GS20 sequencing of a *Manduca sexta *(tobacco hornworm moth) hemocyte cDNA library was provided by Haobo Jiang and is published by Zou *et al *[36]. The *Chrysomela tremulae *(a beetle) midgut GSLFX and *Manduca sexta *midgut are published by Pauchet *et al *[37] and [38] respectively; the GSFLX of cDNA from whole larvae of *Euphydryas aurinia *(marsh fritillary butterfly) was provided by Yannick Pauchet (University of Exeter); the GSFLX-Titanium sequencing of cDNA from wing discs of *Papilio dardanus *(African swallowtail butterfly) by Iva Fuková (University of Exeter); the Sanger capillary and GSFLX data of *Heliconius melpomene *were prepared from wing-discs of developmental stages from late larval through to mid-pupal stage by Ronald Lee and Chris D. Jiggins (University of Cambridge) and is published by Ferguson *et al *[39]. The Sanger capillary and GSFLX data of *Heliconius erato *was also generated from wing-discs and was provided by W. Owen McMillan (North Carolina State University). The GS20 *Melitaea cinxia *dataset from whole larvae is published by Vera *et al *[40] and was downloaded from NCBI's Short Read Archive after communication with Howard Fescemyer (Pennsylvania State University). The *Bicyclus anynana *data are based on the capillary-sequencing technology of a variety of tissues, were obtained from dbEST and is published by Beldade *et al *[41]. The *D. melanogaster *data are also based on capillary-sequencing technology of a variety of tissues and were retrieved from dbEST. We did not include any singletons in the resulting assemblies. For the saturation curves, we used the *H. melpomene *454 preprocessed dataset (without any Sanger sequences) and created pseudo-datasets by randomly splitting it in datasets containing 1/5, 2/5, 3/5 and 4/5 of the initial data. We repeated the procedure 5 times for each pseudo-dataset thus generating and annotating 20 pseudo-assemblies using the same procedures as for the main assembly.

Reference proteomes used in this study were from *D. melanogaster*, *Anopheles gambiae*, *Apis mellifera, B. mori *and *Tribolium castaneum*. For each species we used the RefSeq [42] and UniProt [43] curated proteins, concatenated with the predictions provided by each organism's Genome Database [44-50] and made non-redundant at the 100% level using cd-hit [51]. In the cross-dataset comparison, we identify ORF coverage by calculating the proportion of the reference proteome aligned to the assembly (e-value <= 1e-5; bit-score >= 80 bits) as an indication to gene-finding. We also estimate the proportion of the assembly aligning to the proteome (e-value <= 1e-5; bit-score >= 80 bits) to determine the portion of the assembly likely to be coding and also include the improvement of including a tBLASTx search (e-value <= 1e-15; bit-score >= 80 bits).

### Implementation overview for the biologist

The *est2assembly *platform allows for the processing of data either directly from DNA sequencers (pyrosequencing or Sanger based) or as FASTA files. The software also allows users to combine their own datasets with those available in public databases and a script is included to automatically download such sequences from the European Bioinformatics Institute (EBI). Moreover, any large sequenced genomic regions (such as from Bacterial Artificial Chromosomes; BACs) can contribute CoDing Sequences (CDSs) to the assembler. This form of dataset concatenation has the advantage that a pool of shorter NGS reads will assemble better if a longer sequence (such a full-length mRNA sequence) is also included in the same pool. Currently, two sequencing technologies can be processed: Sanger capillary-based data and 454 pyrosequencing data. The input data is fed into the preprocessest.pl which removes low quality sequences, any adaptors and polyA/Ts that may be present. This script also removes any contaminants and repeats which can cause serious misalignments. Two versions of the processed FASTA files are produced: trimmed and 'masked', the latter accompanied by a quality file and an NCBI Traceinfo XML file which defines trim points in relation to the original files. Researchers can choose to use the untrimmed but masked files or the original sequences (with the XML file which contains the exact regions of high quality sequence) or the trimmed files for assemblers such as Newbler. Further, at this point in the pipeline, graphs can also be generated (using two scripts provided in the distribution) to examine the effect of the pre-processing on the data.

Once a user is satisfied with the quality control of the reads, the assembly can begin. An optimal assembly needs to be chosen according to the needs of a project but often the assembler is used as a black box despite the fact that assemblers are mere computation machines and therefore the results may or may not be biologically meaningful. For this reason, the next step is submitting these files to a second script, parameterize_assembly, which launches the assemblers (currently Newbler and MIRA are supported) with varying parameters, compares the results to one or more reference proteomes and computes a number of indexes suitable for transcriptome projects. Which index is most useful (i.e. the optimality criteria) depends on the aim of the particular project. For example, in a gene-hunting project one may wish to optimize for the number of genes discovered and minimize redundant contigs, where as in SNP project, one may wish to maximize for the number of reads in the assembly and tolerate redundancy which can later be addressed manually for contigs of interest. For the reference proteomes, we suggest that more than one is provided in order to remove species-specific bias and increase the power of detecting coding sequences but, for computational reasons, too divergent species will not be useful for parameterization. In our work and this paper, we used species with a genome sequence which are in the same Phylum as our data datasets. As parameterization is focused on exploring how different parameters behave, the exact details of the reference proteome and BLAST cut-off values are not as important since the platform ensures they are used in a consistent fashion.

Simple BLAST-driven indexes are reported for each proteome: the number of queries which have similarity with a reference protein; the number of reference proteins which have similarity to a contig in the assembly. It then calculates the indexes for each base pair/amino acid rather in order to detect the level of partial ORF sequencing. Further, an annotation redundancy index is based on the number of one-to-many hits (summed in both directions) between the reference proteomes and the assembly. Finally, *est2assembly *reports the number of reads included in the assembly. Which criterion is chosen to identify the best assembly is left to the individual researchers. In this paper, since we are focused on maximizing the 'number of genes found' - like many non-model species projects - we used the reference proteins found as the main criterion.

**Figure 2:**
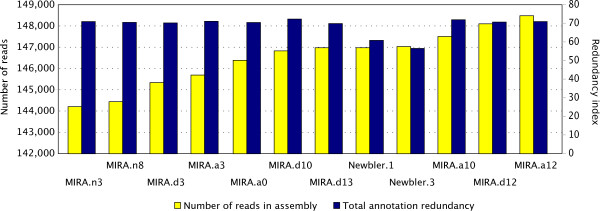
**Exploration of the parameter space on the *E. aurinia *dataset**. Effect of parameterization on assembly is significant. In this dataset, Mira.a0 is the default settings for an 'accurate' assembly. One benchmark is number of reads as lower number of reads result in lower coverage. Another is the redundancy index estimates the level of one-to-many edges (in both directions) exist in an alignment graph between an assembly and the same reference proteome. Newbler seems to outperform MIRA if annotation redundancy is the estimator but see Figure 4.

**Figure 3:**
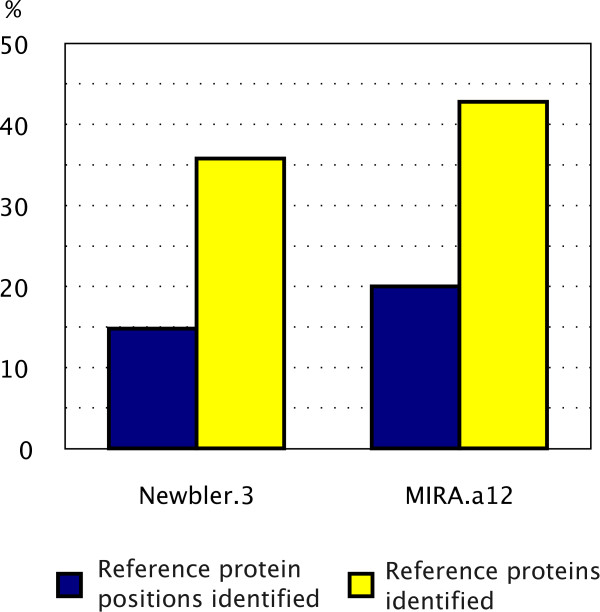
**Comparison of the Newbler.3 and MIRA.a12 assemblers with respect to the numbers of amino acid residues or proteins identified via the *est2assembly *pipeline**. In this *E. aurinia *dataset, we used the BLASTx similarity to *Bombyx mori *(cut-off 50 bits) in order to compare performance. MIRA produces an assembly which identifies more of the reference proteome. Further, at this coverage, we do not have a complete coverage of each gene as the proportion of individual amino acids identified is lower (see text for discussion). As this project is a gene-finding one, we choose the MIRA assembly for downstream application.

Ignoring the parameterization step is not recommended, as Figure 2 shows: the MIRA.a0 parameter set is the default for MIRA's 'accurate quality' setting but produces a suboptimal assembly using the criteria of annotation redundancy. One should note that even though transcriptome sequencing (especially with NGS technologies) is producing transcripts from the whole mRNA, it is unlikely that full length transcripts are sequenced. Figure 3 shows that proportion of identified proteins in terms of CDS coverage was significantly lower than the proportion in terms of number of identified proteins (e.g. for MIRA 20% vs 42%). In addition, for this dataset, MIRA outperformed Newbler with our chosen criterion, showing it is important to attempt an assembly with more than one assembler.

Once the desired assembly is chosen, users may opt for the removal of a subset of redundant contigs using trim_assembly.pl and evaluate if there is a loss of 'number of genes found'. By redundancy we mean here the fraction of contigs that are likely to originate from the same locus - as defined by the degree of similarity. Often, an assembler fails to align them due to a high number of mismatches caused by a non-conserved region (such as in non-coding regions) accumulating mutations, an alternative exon or - in libraries constructed from outbred individuals- multiple SNPs. The aim of a reduction of redundancy is to reduce the strain of computing resources on the subsequent annotation steps. Researchers will, therefore, annotate a considerably smaller dataset. We support GFF conversion for a number of publicly available tools that we consider to be of most use in transcriptome project and we use routinely: the CAF format (the new standardized file format for assemblies); prot4EST (ORF prediction); BLAST (similarity annotation); annot8r (Gene Ontology, Enzyme Commission and KEGG pathway term assignment according to similarity to known proteins) and InterProScan (protein domain identification).

As our interest is primarily in ensuring that data produced by multiple researchers can be integrated, we decided to utilize the community tools of BioPerl and GMOD. The bioinformatics community has been converging on a set of standard formats. One such flat-file format for sequence annotation is the General Feature Format (GFF) specification which is currently being standardized as version 3. The GFF format is a tab and semicolon delimited file making it both machine- and spreadsheet- readable and has become the format of choice for the GMOD software group. From a database perspective, there are two additional important formats: BioPerl's Bio::DB::SeqFeature and Chado. The former is a highly denormalized database schema which allows for rapid queries of sequence data by sacrificing control of data integrity. Chado, on the other hand, is a normalized modular database schema created to serve as the main data warehouse of multiple types of data. It is logical therefore for researchers to utilize Chado as a data warehouse and Bio::DB::SeqFeature for driving user-visualization software such as GBrowse. Data can be loaded into a database via BioPerl and we provide a script to load multiple GFFs in the correct order and allow for later additions. The Chado schema requires a PostgreSQL database and we find that the SeqFeature database works well with PostgreSQL as well. Once the database is loaded, one can use to drive popular tools such as GBrowse, Apollo and Artemis (Figure 1B) in order to curate the project. Transcriptome project curation requires the ability to join contigs and we find that the user-friendliness of the proprietary program Geneious (Biomatters Ltd, Auckland New Zealand) is efficient for the purpose and a free version is available. A future version of this platform may make use of Geneious' interactivity interface (API). Due to the popularity of GBrowse as a sequence viewer, we provide configuration files that can be readily customized. When the complete analysis is loaded, researchers and their collaborators can view any annotated contig in three inter-linked web pages: assembled contig, predicted ORF and protein (Figure 1C).

## Results

As est2assembly is unique in the field as it is not one pipeline but a complete framework to analyze transcriptomes from raw data to a format wet-lab biologists can analyze. The preprocessing step has been built to take advantage of NCBI's TraceXML in order to annotate vector and clipping positions. This allows us to use the original (unmasked) data to produce the assembly using MIRA which can make intelligent decisions if a clipped region is a false positive. The assembler parameterization allows bioinformaticians to seamlessly explore the parameter space as Figures 2 and 3 mentioned above (Implementation overview for the Biologist). Had we used the default settings, we would have an assembly that had a lower number of identified reference proteins (data not shown), a lower number of reads used but similar degree of the redundancy index (Figure 2). Likewise, had we opted for the Newbler assembler without investigating the MIRA assembly, we would have both a lower number of proteins identified and shorter CDSs (Figure 3).

In addition, as we mention above and show below, current NGS assemblers produce non-redundant contig sets. This is a correct procedure in order to avoid assembling close paralogs (as joining contigs is easier that splitting them) but results in a downstream computational problem with annotating a redundant set of objects. Our trim_assembly approach is highly customizable using concepts intuitive to biologists and produces better clustering than the standard cd-hit-est we used to use: for one of the more redundant assemblies we started with 54,748 contigs and reduced it to 37,012 contigs with trim_assembly when compared to 51,012 when using cd-hit-est with both strand search enabled.

Subsequently, we extend the usefulness of prot4EST by allowing users to build a ORF model even for species where no ORF is annotated in the public domain. Further, our SNP pipeline uses a similar approach as SEAN but predicts more markers (Table 1) and is built to be fast and efficient with a large number of data (e.g. identification and ORF classification of SNPs in an assembly needs ca. 60 minutes for 14,817 contigs with 246,477 sequences when SEAN needed more than one day due to high I/O usage) but also to decrease the number of SNPs which would be useful to wet-lab biologists: with the 454 technology we have more candidate SNPs that biologists can afford to screen or make use of (see Table 1 for comparison with MIRA which is a liberal predictor). Manual inspection is essential in order to identify markers which are most useful in downstream genotyping methods. For example, any base covered with less than 4 reads is of no use for a SNP call as one cannot distinguish a SNP from a sequencing error. In addition, the platform predicts high quality SNPs by demanding a certain region surrounding the SNP to be invariable in order to assist with primer design. Further, by comparing with the position in the predicted ORF, a marker is classified as non-coding/coding and then determined if causing a synonymous (amino acid is preserved) or non-synonymous (amino acid is changed) mutation. Perhaps, however, the single most important innovation in the field of transcriptome processing is the utilization of the GFF file format and integrating the assembly, protein predictions and annotations into a format the GMOD framework.

**Table 1:** SNP marker identification in trimmed assemblies

Dataset	Trimmed contigs	Total High Quality SNPs	Coding SNPs	Synonymous SNPs	Predicted with SEAN	Predicted with MIRA
*D. melanogaster *(Sanger)	24,629	77,969	49,341	15,535	8,060	415,501

*B. anynana *(Sanger)	11,942	18,271	10,783	4,773	3,282	16,847

*M. cinxia *(GS20)	12,492	5,622	3,521	1,979	5,918	Not estimated

*M. sexta *(Sanger + GSFLX)	12,635	7,593	5,026	2,594	6,510	527,469

*C. tremulae *(GSFLX)	9,771	3,238	2,087	978	2,905	Not estimated

*E. aurinia *(GSFLX)	8,984	6,132	3,921	2,170	5,543	Not estimated

*H. erato *(Sanger + GSFLX)	12,130	8,720	4,893	2660	8,744	22939

*H. melpomene *(Sanger + GSFLX)	16,631	65,047	28,536	18,613	27,526	4,090,305

*P. dardanus *(GSFLX Titan.)	25,083	49,421	20,694	11,069	7,061	Not estimated

### Utility

**Figure 4:**
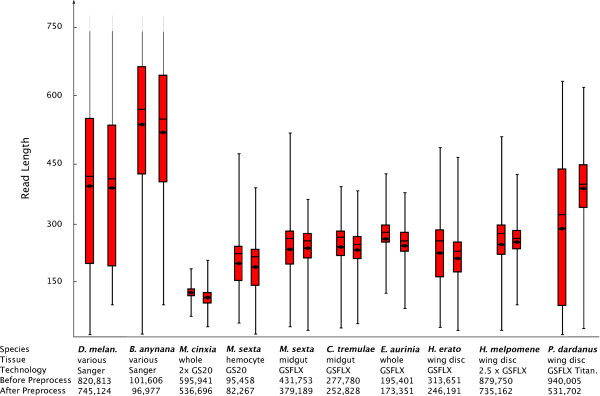
**Boxplot of read length before and after pre-processing for each dataset, showing 25% and 75% intervals, the horizontal bar shows the median, the diamond shows the mean, whiskers encompass entire data range**. Such information offers an overall picture of a sequencing run's quality.

We used a diverse dataset to test and build this platform and due to the standardized approach which it follows, we have been able to evaluate data from different sequencing technologies and protocols in order to offer insights on non-model species transcriptomics. The cost-effectiveness of NGS has been a primary motivation for non-model species researchers to initiate project yet others are worried about the quality of data resulting from such short reads. Examination of the data after pre-processing is essential in order to make a meaningful comparison and our graphical tools allows researchers to compare their raw data with other datasets (Figure 4).

**Figure 5:**
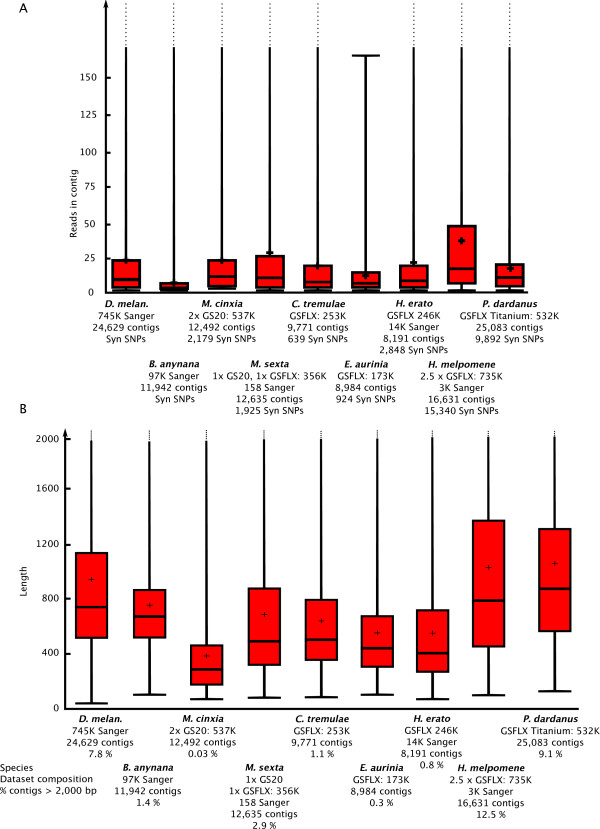
**Boxplot of (A) number of reads assembled in each contig and (B) contig length for each dataset, showing 25% and 75% intervals, the horizontal bar shows the median, the plus sign shows the mean, whiskers encompass entire data range**. Sanger technology has been considered a cleaner technique despite a higher cost but the B. anynana dataset (ca 97K sequences) performs poorly when compared to GSFLX. The earlier GS20 technology is significantly inferior and of limited use in transcriptome sequencing.

In gene discovery projects, if one hopes to provide an accurate level of annotation, the length of the contigs is an important element which must be considered during project design. As Figure 5 shows, the number of reads increases significantly the length of contigs (for example when comparing *H. melpomene *with all the other GSFLX datasets) but technology has the largest effect. GS20 seems to be of limited use and the newest GSFLX-Titanium has comparable contig length to 2.5 GSFLX runs. Further, with increased read number and length, we get an increased contig coverage. The coverage of each contig (how many sequencing reads are assembled together in one contig) is an important limiting factor in SNP prediction, error correction and the overall quality of the assembly. For population genomicists, substantial contig coverage offers an additional advantage in being able to estimate the frequency of a particular SNP marker. Further, low frequency non-synonymous SNPs can guide a curator to regions of misassembly or erroneous ORF prediction. Once verified to be true non-synonymous SNPs, curators can look for genes showing an excess of non-synonymous polymorphisms and, therefore, possibly evolve under balancing selection. Even though, current users need to perform this latter step manually, it would be of use to automate it for assemblies which are hand-curated. For such an investigation to be profitable, however, the design of the project, especially how many and which individuals, to be included in the cDNA library must be carefully planned.

The number of contigs is, however, rarely an accurate prediction of the number of genes sequenced. Non-coding DNA (e.g. UTR - UnTranslated Region or intron read-throughs) sequenced from multiple haplotypes is not easy to assemble due to high levels of heterozygosity caused by the fact that the majority of the UTR is evolving without constraints. Upon investigation, this seems to be the major cause of contig inflation and the platform can use two methods to alleviate the issue by filtering in favor of regions likely to be coding. One method is used in Figure 5 but we may have the unfortunate effect of removing small contigs containing novel proteins which are small in size and low in expression. The other method - available only to users with a dataset from related species - is to use a tBLASTx approach (via analyse_assembly.pl) to complement the BLASTx approach of the reference proteome. Novel proteins, even if evolving rapidly, are expected to show significant similarity on the amino acid level between the two species. The platform allows one to extract the contig regions matching this coding fraction and thus have a dataset known to be coding. The two approaches are not mutually exclusive but can be complementary: the trim_assembly.pl is highly customizable regarding similarity and abundance levels whereas the tBLASTx approach will not tackle the issue of redundancy (i.e. two contigs originating from one locus).

**Figure 6:**
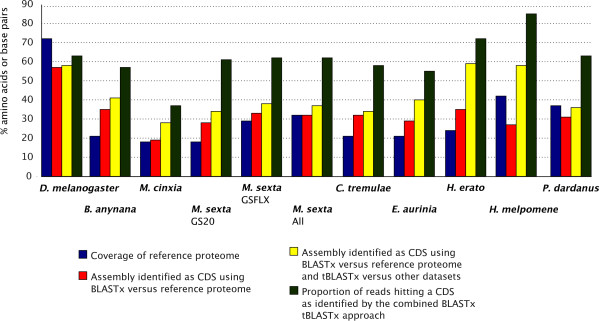
**Comparison of the number of genes and proteins identified using different 454 based sequencing technologies (GS20, GSFLX and GSFLX-Titanium)**. For each dataset, the accuracy of the results depends on how similar the target and reference transcriptomes are and the improvement with tBLASTx is an indication of novel protein data supported by at least two species. Such cases warrant a more thorough investigation and can result in the determination of taxon specific- or rapidly evolving genes. The proportion of reads from the sequencer (after pre-processing) which are part of these coding regions is also shown. This can guide future project designs which wish to aim to alter the representation of non-coding in the sequenced sample.

We can, therefore, conclude that a better assembly benchmark is the identification of proteins from a reference proteome and the portion of the assembly identified as coding (CDS; CoDing Sequence). The quality of a sequencing experiment can thus be evaluated by extracting the CDS fraction and then calculating the proportion of reads contributing to this portion of the assembly (e.g. by doing a BLAST similarity search). In our dataset, we find that the *Drosophila *dataset covers only 70% of the *Drosophila *proteome and only 56% of the assembly is defined as coding (Figure 6). From the BioMart.org website we calculated that the proportion of non-UTR in annotated *D. melanogaster *genes is only 80%. The trend of having a significantly lower CDS proportion than expected is maintained across the data when a BLASTx versus a reference proteome is used. One reason can be purely technical: in a dataset originating from a library with large number of haplotypes, there is a contig inflation when the UTR is included for assembly. This effect is more likely to be present in non-model species where isogenic lines or sufficient levels of inbreeding are unattainable. The second technical issue, especially in NGS datasets, is that due to the fragmentation step involved, there can be a preference for sequencing of the transcript ends and therefore UTR [52].

**Figure 7:**
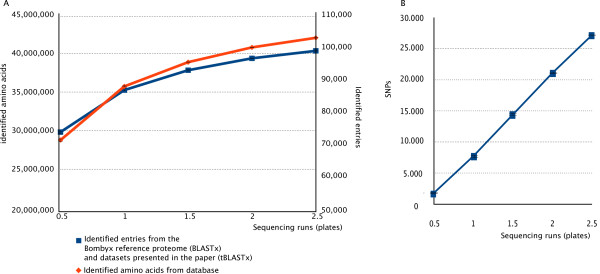
**Saturation curve of 454 GSFLX sequencing using the *H. melpomene *dataset**. The error bars show the min/max of each data point as verified with 5 independent pseudo-samples. (A) Researchers can obtain a substantial number of genes with data from one half-plate with saturation for the transcriptome of this sample near the 2.5 plates. (B) SNP marker identification is linear in this dataset with an average of 1,757 high quality SNPs identified in one half-plate.

Regardless whether a measure against redundancy is used, if multiple datasets are available, one can explore whether a reference proteome is useful and whether using a phylogenetic framework assists in gene-finding. Due to the taxon focus and species richness in our data, we expect that mutual tBLASTx searches among the assembled NGS datasets (excluding the species used a query) as reference databases, will identify additional proteins which may be absent from the reference proteome, or sufficiently diverged to be missed by comparison to it. Because the *B. anynana*, *M. cinxia*, *E. aurinia*, *P. dardanus*, *H. erato *and *H. melpomene *datasets originate from butterflies with the latter two being from the same genus (*Heliconius*) and the *M. sexta *datasets originate from a moth in the same superfamily as the reference proteome *B. mori *[53] we expect and find that the butterflies will show a significantly higher improvement with tBLASTx than *M. sexta*. Oddly, we also find a large improvement in *C. tremulae*, a beetle which uses the *Tribolium castaneum *as a reference but are in different superfamilies. This improvement is unlikely to be due to a poorer annotation in *Tribolium *versus *Bombyx *as the reference annotation proportions are relatively similar in all non-model species datasets. It is not unlikely that *Tribolium *is not a good model for *C. tremulae *especially if one begins to consider the difference in their ecology. The other, not mutually exclusive, possibility is that there is a significant degree of rapidly evolving proteins in these non-model species. Nevertheless, via the tBLASTx approach, each of the *Heliconius *data has now a CDS proportion comparable to the *Drosophila *gold standard. Indeed, by also counting the number of sequence reads belonging to the estimated CDS (Figure 7) we are able to see that the *Heliconius *datasets have also a higher proportion of reads belonging to the CDS. This observation provides some evidence that contig inflation in this case is more likely to be driven by unassembled UTR rather than due to fragmentation of the transcript ends.

One other point of note is relating to the procedure of choosing a cDNA generation protocol. All NGS datasets compared here use the SMART technology to produce cDNA apart from the GS20 dataset of *M. sexta *which was produced using GC-rich random primers. We do not know why the number of reads was much lower than the GS20 dataset from *M. cinxia *but a significantly higher proportion of the reads matches a predicted CDS which translates to a better return to projects aimed at gene-hunting. We cannot be sure why this occurs: it could be due to a high number of low quality reads in *M. cinxia*, it could be due to a slightly better protein identification based on the closely related *B. mori *but it is logical to expect that, in species known to have a GC enriched coding sequences, the primer protocol would enrich for regions with high GC content and therefore more likely target coding regions.

Finally, researchers often wish to know how deep one should sequence in order to sequence the complete transcriptome in their sample. This is important in planning non-model species transcriptomes projects. As the *H. melpomene *454 data originate from 2.5 full-plates (i.e. 5 half-plates) and the cDNA is harvested from a single tissue (wing discs) we used 20 pseudo-assemblies to investigate the effect of deeper sequencing. As Figure 7 shows, most transcripts for that particular tissue were identified after 1.5 half plates were sequenced as the exponential curve is approaching a plateau (Figure 7A). With 1 half-plate, however, 74.5% of the plateau value for proteins identified was attained, showing that even shallow sequencing of a non-model species is highly worthwhile. For SNP detection, the function is not exponential (Figure 7B): with each subsequent run the number of high quality SNP markers increased linearly.

## Discussion

There are several advantages of *est2assembly *over other platforms for processing EST raw data (e.g. [54,55]). First, preprocessing of raw sequence is essential and our platform offers a standard method for consistently accomplishing this for hundreds of thousands of sequences with straightforward user customization. Second, parameterizing an assembler is a tedious process and our platform is the only one which automates many of the routines. Third, annotation of an assembly with *est2assembly *can be readily standardized and automated for processing large numbers of datasets with minimum investment in time. Deciding on the optimal assembly is a subjective process and depends on the project but by providing the means to explore the parameter space allows for a standardization of an approach which is often ad-hoc. We calculate the BLAST-based index using two approaches and can determine the number of unique proteins found, actual proportion of amino acids found and obtain an estimate of the assembly proportion that is actually coding. In this case, as more datasets are published, we can benchmark laboratory protocols and sequencing technologies involved in acquiring full length genes. Importantly, the rich log output guides the wet-lab biologist who generated the data to perform in-depth investigations and hold a better understanding of their project. With *est2assembly*, we have not aimed to produce a 'black box' but a program which gives feedback to the user as to the quality and characteristics of the different assemblies achieved with their data. We showed that analysis of an assembly can give important insights to the technologies and protocols employed to acquire a transcriptome. Future work can focus on including more annotation modules and developing a Java/JDBC-driven Graphical User Interface (GUI) and relational database to allow molecular biologists with no computing knowledge to supervise the data analysis.

Shallow sequencing EST projects are becoming a gold-mine for biologists working on non-model species and are often used for both gene or SNP discovery but until now no software exists to link the SNP to both an assembly and the codon it may be part of. The *est2assembly *platform allows for the classification and identification of SNPs which may be under selection or point to a misalignment. Such data are important in manual curation of an assembly and lacking from any other software. We can also obtain coding synonymous SNPs for which a PCR primer is straightforward to design but are under low levels of selection. Non-coding markers are also useful to researchers who wish to investigate selection in non-coding DNA.

Special considerations, however, have to apply to projects working on non-model species, especially when datasets are restricted for financial or biological reasons. Often the design of the experiment is not conceived with full knowledge of a technology's capabilities and limitations. Here we show that different technologies and lab protocols differ in their ability to produce an assembly and project design plays an important role. At times, but not always, such project design bottlenecks can be overcome. For example, assemblers treat the common issue problem of inflated contig number caused by non-optimal alignments by assuming that they are based solely on sequencing errors or repeats. The result is that fewer genes are discovered in non-model species. The issue is confounded because researchers undertake a transcriptome project for different reasons. Even though in gene discovery project the norm is to sequence a cDNA library from specific tissues and with a limited number of haplotypes, this is rarely the case in most EST projects of non-model species. Researchers often utilize EST projects as both a gene-finding project and a SNP discovery protocol and therefore are tempted to include a high number of out-bred individuals. It should be noted that both Newbler and MIRA are not clustering algorithms but assemblers and therefore their main aim is not to identify alternative splicing events or cope with a high degree of heterozygosity in the sequenced sample. There are methods to alleviate the problem such as including a final clustering step (e.g. miraEST [56]; CLOBB [57]; or CAP3 [58]. Our platform does not yet contain such a clustering step as the levels of heterozygosity can vary and a supervised algorithm we are developing as a future module may provide a more optimal solution. Such an algorithm would be tailored (i.e. trained) for each transcriptome project and make assignment of supercontigs (e.g. the merging of alternative splicing events and non-coding regions belonging to the same locus) more robust. Another issue which cannot be resolved using bioinformatics is the quality of material used for cDNA preparation. Even though we cannot be certain regarding the cause, the *M. cinxtia *and *E. aurinia *datasets argue against a whole animal approach in constructing the cDNA library. Such cases have been shown to be problematic in enzymatic reactions due to PCR-inhibiting pigments [59]. The inclusion of micro organisms in the digestive tract or the outer body can also result in acquiring contaminating sequence from another species. The later cases can be investigated bioinformatically [60,61] so as to prevent generating an erroneous transcriptome survey.

## Conclusion

In conclusion, the modern transcriptome sequencing approaches are very powerful and cost effective but they still yield partial transcriptomes. In the future, however, our single most important limitation will not be raw transcriptomic or genomic data. We have shown that the ability to accurately annotate an assembly depends on using a correct reference proteome or utilize phylogenetic framework. Further, comparison to an appropriate reference proteome is invaluable in choosing among different assemblies, yet such proteomes are themselves incomplete. A concerted annotation effort based on transcriptome sequencing from a diverse phylogenetic collection is required, which will accelerate the filling of proteome space beyond the limited set of model organisms that currently occupy it. With the NGS capabilities, it is obvious that such an effort should make full use of transcriptomic data in order but we still lack the necessary infrastructure. The *est2assembly *software, however, has enabled the development of such infrastructure, an alpha stage preview of which is available at http://www.insectacentral.org.

## Availability and requirements

Project name: *est2assembly*

Project home page: http://code.google.com/p/est2assembly/

Operating system: Linux

Programming language: Perl

Dependencies on proprietary software: None

Other requirements: NCBI-BLAST, EMBOSS toolkit, BioPerl dev-branch, PostgreSQL, MIRA (included), sff_extract (included)

Optional programs (included): annot8r, prot4EST

License: General Public License version 3

## Abbreviations

BAC: Bacterial Artificial Chromosome; CDS: CoDing Sequence; EBI: European Bioinformatics Institute; EST: Expressed Sequence Tag; GFF: General Feature Format; GMOD: Generic Model Organism Database; GPL: General Public License; GUI: Graphical User Interface; NGS: Next Generation Sequencing (technology); ORF: Open Reading Frame; PAS: PolyAdenylation Signal; SNP: Single Nucleotide Polymorphism; UTR: UnTranslated Region.

## Authors' contributions

AP conceived, designed and performed the study; analyzed and interpreted data; coded the software and drafted the manuscript. RS co-authored the GFF writing software and the GBrowse schema. RHfC and DGH drafted the manuscript, financed and provided infrastructure for the study. All authors approved the final version of the manuscript.
